# Inactivation of Presenilin in inhibitory neurons results in decreased GABAergic responses and enhanced synaptic plasticity

**DOI:** 10.1186/s13041-021-00796-5

**Published:** 2021-05-25

**Authors:** Sang Hun Lee, Vadim Y. Bolshakov, Jie Shen

**Affiliations:** 1 Department of Neurology, Brigham and Women’s Hospital, Harvard Medical School, Boston, MA 02115 USA; 2 Department of Psychiatry, McLean Hospital, Harvard Medical School, Belmont, MA 02478 USA; 3 Program in Neuroscience, Harvard Medical School, Boston, MA 02115 USA

**Keywords:** Alzheimer’s disease, Interneuron, Conditional knockout, Hippocampus, Schaffer collateral, Short-term synaptic plasticity, Long-term potentiation, Calcium homeostasis

## Abstract

Mutations in the *Presenilin* genes are the major genetic cause of Alzheimer's disease (AD). Presenilin (PS) is highly expressed in the hippocampus, which is particularly vulnerable in AD. Previous studies of PS function in the hippocampus, however, focused exclusively on excitatory neurons. Whether PS regulates inhibitory neuronal function remained unknown. In the current study, we investigate PS function in GABAergic neurons by performing whole-cell and field-potential electrophysiological recordings using acute hippocampal slices from inhibitory neuron-specific *PS* conditional double knockout (IN-*PS* cDKO) mice at 2 months of age, before the onset of age-dependent loss of interneurons. We found that the frequency of spontaneous inhibitory postsynaptic currents (sIPSCs) is reduced in hippocampal CA1 neurons of IN-*PS* cDKO mice, whereas the amplitude of sIPSCs is normal. Moreover, the efficacy of inhibitory neurotransmission as assessed with synaptic input/output relations for evoked mono- and di-synaptic IPSCs is markedly lowered in hippocampal CA1 neurons of IN-*PS* cDKO mice. Consistent with these findings, IN-*PS* cDKO mice display enhanced paired-pulse facilitation, frequency facilitation and long-term potentiation in the Schaffer collateral-CA1 pathway. Interestingly, depletion of intracellular Ca^2+^ stores by inhibition of sarcoendoplasmic reticulum Ca^2+^ ATPase results in a reduction of IPSC amplitude in control hippocampal neurons but not in IN-*PS* cDKO neurons, suggesting that impaired intracellular calcium homeostasis in the absence of PS may contribute to the deficiencies in inhibitory neurotransmission. Furthermore, the amplitude of IPSCs induced by short trains of presynaptic stimulation and paired-pulse ratio are decreased in IN-*PS* cDKO mice. These findings show that inactivation of PS in interneurons results in decreased GABAergic responses and enhanced synaptic plasticity in the hippocampus, providing additional evidence for the importance of PS in the regulation of synaptic plasticity and calcium homeostasis.

**Supplementary Information:**

The online version contains supplementary material available at 10.1186/s13041-021-00796-5.

## Introduction

Alzheimer’s disease (AD) is the most common neurodegenerative disorder characterized by progressive memory loss and cognitive decline. Mutations in the *Presenilin* genes account for ~ 90% of all identified causative mutations in familial AD, highlighting their importance in AD pathogenesis. Genetic and electrophysiological studies demonstrated that Presenilin (PS) in excitatory neurons plays an essential role in learning and memory, synaptic transmission and plasticity, and their age-related survival in the cerebral cortex [1–9].

The optimal balance between excitatory and inhibitory neurotransmission is essential for the function of local neuronal networks [10]. Previous studies showed that an imbalance between excitatory and inhibitory signals in the hippocampus may contribute to cognitive impairment in AD patients [11–13]. For example, AD patients and young carriers of apolipoprotein E4 alleles exhibited abnormal hippocampal overactivity and GABAergic interneuron dysfunction [14–18]. Overactive neural circuits have also been reported in various APP transgenic mouse models [19–23]. However, it remained unclear whether PS is required for normal function of interneurons.

We recently generated interneuron-specific *PS* conditional double knockout (IN-*PS* cDKO) mice using the *GAD2-IRES-Cre* driver, in which Cre is expressed under the control of the endogenous *GAD2* promoter [24]. We discovered that selective inactivation of PS in interneurons results in age-dependent loss of cortical interneurons and increases of apoptotic interneurons as well as astrogliosis and microgliosis in the cerebral cortex [24]. In the present study, we investigate the role of PS in interneurons in the local circuit of the hippocampal Schaffer collateral (SC)-CA1 pathway using IN-*PS* cDKO mice at 2 months of age, before the onset of interneuron loss in the cerebral cortex [24]. We found that the frequency of spontaneous inhibitory postsynaptic currents (sIPSCs) is reduced in CA1 neurons of IN-*PS* cDKO mice. Moreover, synaptic efficacy at the level of input/output relations for evoked mono- and di-synaptic IPSCs is markedly lowered in IN-*PS* cDKO mice. Furthermore, IN-*PS* cDKO mice exhibit enhanced paired-pulse facilitation, frequency facilitation and long-term potentiation in the SC synapses whereas excitatory AMPA receptor-mediated basal synaptic transmission is normal. Importantly, the increase of the evoked IPSC amplitude in the course of repetitive presynaptic stimulation is impaired in CA1 neurons of IN-*PS* cDKO mice, and blockade of SERCA mimics and occludes the GABAergic IPSC deficits in IN-*PS* cDKO CA1 neurons. Taken together, our findings demonstrate that selective inactivation of PS in interneurons results in impaired GABAergic inhibition and increased excitability of hippocampal CA1 neurons in IN-*PS* cDKO mice.

## Methods

### Generation of inhibitory neuron-specific *PS* cDKO Mice

The generation of inhibitory neuron-specific *PS* cDKO mice has been previously described [24]. Briefly, *fPS1/*+*; PS2*+*/− ; GAD2-IRES-Cre/*+ mice were bred with *fPS1/fPS1; PS2−/− *mice to obtain *fPS1/fPS1; PS2−/−; GAD2-IRES-Cre/*+ (IN-*PS*cDKO) mice. IN-*PS*cDKO and littermate control (*fPS1/fPS1; PS2−/−*) mice used in the study were obtained from breeding *fPS1/fPS1; PS2−/−*mice together with *fPS1/* + *; PS2−/−; GAD2-IRES-Cre/*+ mice.

All mice were housed in humidity- and temperature-controlled rooms maintained on a 12:12 h light: dark cycle and were given standard rodent chow and water. IN-*PS*cDKO and littermate control mice were maintained in the C57BL/6 J 129 hybrid genetic background. All procedures were approved by the IACUC committees of Harvard Medical School and Brigham and Women’s Hospital, and conform to the USDA Animal Welfare Act, PHS Policy on Humane Care and Use of Laboratory Animals, the “ILAR Guide for the Care and Use of Laboratory Animals” and other applicable laws and regulations.

### Preparation of brain slices for electrophysiology

Hippocampal slices were prepared from both male and female IN-*PS* cDKO and littermate control mice at 2 months of age. Mice were decapitated after being anesthetized with ketamine (100 mg/kg) + xylazine (10 mg/kg) + acepromazine (3 mg/kg). The brain was removed and placed in ice-cold (4 °C) oxygenated (95% O_2_/5% CO_2_) high sucrose and magnesium solution containing (in mM) the following: 200 Sucrose, 25 NaHCO_3_, 10 Glucose, 3 KCl, 1.25 NaH_2_PO_4_, 1.2 Na-pyruvate and 0.4 Na-ascorbate, 7 MgCl_2_, and 0.5 CaCl_2_. Horizontal hippocampal slices (400 μm thick) were prepared using a vibratome (VT1200S, Leica, Germany), and transferred to an incubation chamber having oxygenated artificial cerebrospinal fluid (ACSF) containing (in mM) the following: 125 NaCl, 3 KCl, 1.25 NaH_2_PO_4_, 1 MgCl_2_, 2 CaCl_2_, 25 NaHCO_3_, 10 Glucose, 1.2 Na-pyruvate and 0.4 Na-ascorbate, adjusted to 310 ± 5 mOsm (pH 7.4). The slices were allowed to recover at 34 °C for 1 h and then placed in a recording chamber constantly perfused with heated ACSF (30 ± 1 °C) and gassed continuously with 95% O_2_ and 5% CO_2_. The flow rates of bathing solution and the volume of the recording chamber for slices were 2.2 ml/min and 1.2 ml, respectively. Hippocampal slices were visualized using an upright microscope equipped with differential interference contrast (DIC) optics (BX51WI, Olympus, Japan). The DIC optics was used for visualization of neurons in the course of whole-cell recordings. In a subset of experiments, the following drugs were used at the following concentrations via bath application or adding intracellular recording solutions: Picrotoxin (100 µM, Tocris #1128), Bicuculline methochloride (20 µM, Tocris #0131), D-AP5 (50 µM, Tocris #0106), NBQX disodium salt (10 μM, Tocris #1044), QX314 chloride (5 mM, Tocris #2313) and Thapsigargin (2 μM, Sigma-Aldrich #T9033).

### Electrophysiological analysis

For whole-cell patch clamp experiments, spontaneous inhibitory postsynaptic currents (sIPSCs) were recorded from CA1 pyramidal neurons in voltage-clamp mode at a holding potential of − 70 mV in the presence of blockers of AMPA (10 µM NBQX) and NMDA (50 µM AP5) receptors. Monosynaptic IPSCs for the input/output relations were elicited by the stimulation electrode placed close to the recording electrode in the CA1 stratum pyramidale (~ 100 μm), and recorded at a holding potential of − 70 mV in the presence of blockers of AMPA (10 µM NBQX) and NMDA (50 µM AP5) receptors. The stimulation pulses ranging from 30 to 150 µA were delivered using a stimulus isolation unit (A365, World Precision Instruments, USA) with an unipolar metal microelectrode. The recording pipettes (3–5 MΩ) were filled with a solution containing (in mM) the following: 130 KCl, 10 phosphocreatine, 20 HEPES, 4 MgATP, 0.3 NaGTP, 5 QX314 and 0.2 EGTA with the pH adjusted to 7.30 with KOH (295–300 mOsm). To obtain biphasic responses consisting of the IPSC/EPSC sequences, the stimulation electrode was placed away from the recording electrode in the CA1 stratum pyramidale (200–300 μm), and then the IPSCs were recorded at a holding potential of 0 mV and EPSCs were recorded at a holding potential of −75 mV with low [Cl^−^] Cs^+^-based pipette solution. The intracellular solution in these experiments contained (in mM) the following: 120 Cs-methanesulfonate, 20 tetraethylammonium-chloride, 20 HEPES, 4 MgATP, and 0.3 NaGTP, 5 QX314 and 0.2 EGTA with the pH adjusted to 7.30 with CsOH (295–300 mOsm). The series resistance (Rs) after establishing whole-cell configuration was between 15 and 25 MΩ. EPSC recordings with > 20% series resistance changes were excluded from the data analysis.

For extracellular field recordings, stimulation pulses were delivered with a stimulus isolation unit (A365, World Precision Instruments, USA) using a unipolar metal stimulation microelectrode. Field excitatory postsynaptic potentials (fEPSPs) were recorded in current-clamp mode with ACSF-filled patch pipettes (1.5–2 MΩ). All fEPSPs were recorded with a stimulation strength that yielded ~ 50% of the maximal response. Data were collected with a MultiClamp 700B amplifier (Molecular Devices, USA) and digitized at 10 kHz using the A/D converter DIGIDATA 1322A (Molecular Devices, USA). Data were acquired and analyzed using a custom program written with Igor Pro software (Version 6.3; Wave-Metrics) and Clampfit (Version 10.3; Molecular device).

For AMPA receptor-mediated input/output (I/O) curves, I/O relations were obtained by plotting the amplitude of fiber volley (FV) versus the fEPSP slope in the presence of blockers of NMDA (50 µM AP5) and GABA_A_ receptors (100 µM Picrotoxin). 10 traces were averaged for each stimulation intensity, and the amplitude of the FV was measured relative to the slope of the fEPSP. The stimulation rate was 0.2 Hz. The average linear fit slope was calculated to obtain I/O relationships for each slice tested. In LTP recordings, after baseline responses were collected every 15 s for 15 min, LTP was induced by five episodes of theta burst stimulation (TBS) delivered at 0.1 Hz. Each episode contained ten stimulus trains (5 pulses at 100 Hz) delivered at 5 Hz. To generate summary graphs (mean ± SEM), individual experiments were normalized to the baseline, and four consecutive responses were averaged to generate 1 min bins. These were then averaged together to generate the final summary graphs. Paired-pulse facilitation (PPF) was measured as the ratio of the second fEPSP slope relative to the first fEPSP slope, evoked by two identical presynaptic stimuli. Synaptic facilitation was measured as the percentage of the fEPSP slope versus the first fEPSP slope at a given stimulus train in individual slices.

### Data quantification and statistical analysis

Data acquisition and quantification were performed in a genotype blind manner. All statistical analysis was performed using Prism (Version 9; GraphPad software), Excel (Microsoft), Igor Pro (Version 6.3; Wave-Metrics) or Clampfit (Version 10.3; Molecular device). All data are presented as the mean ± SEM. The exact sample size (e.g. the number of mice, brain slices, brains or neurons) of each experiment is indicated in the figure.

**Fig. 1:**
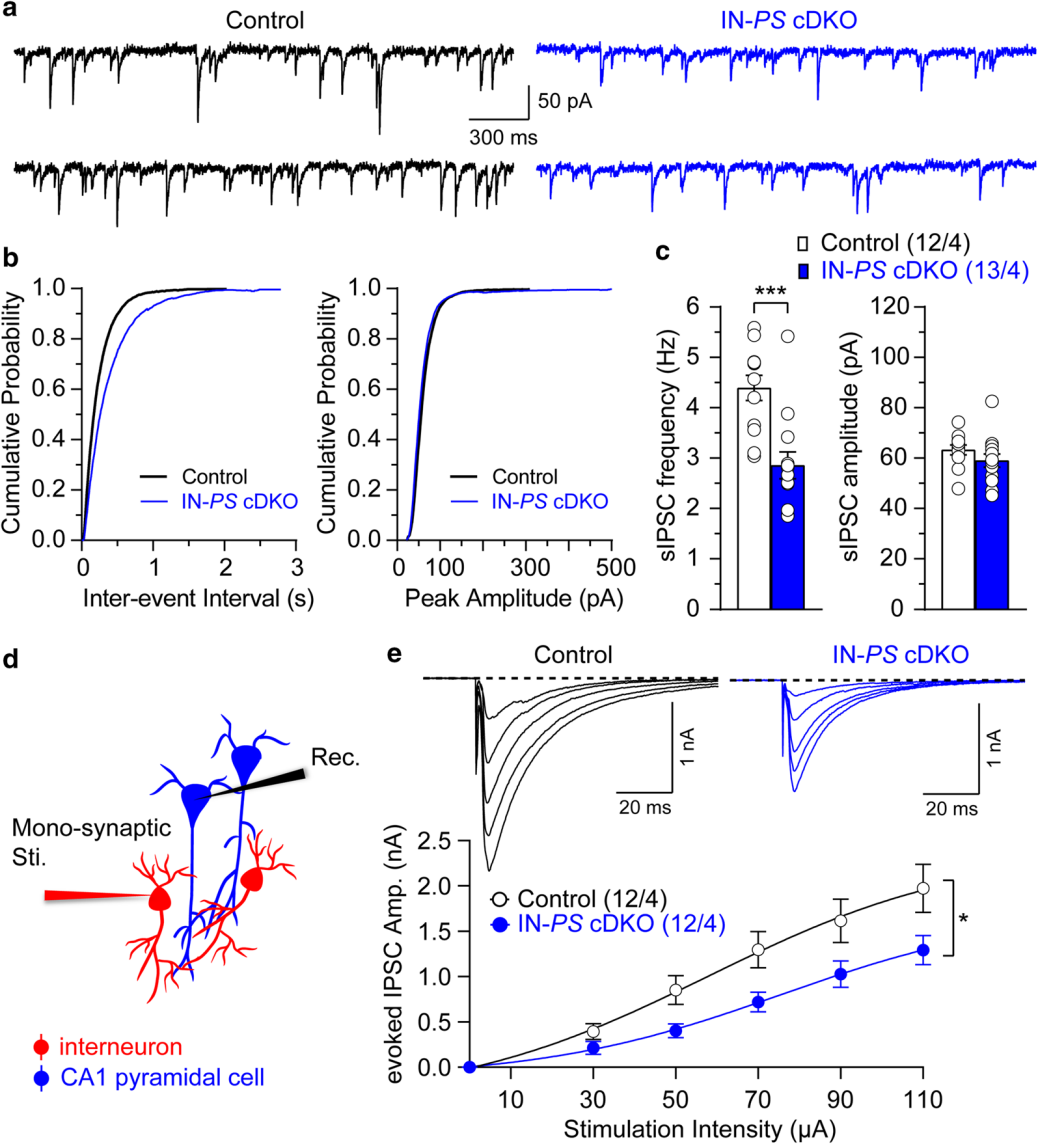
Decreased GABAergic synaptic responses in hippocampal CA1 neurons of IN-*PS* cDKO mice. **a** Representative spontaneous inhibitory postsynaptic currents (sIPSCs) recorded in CA1 pyramidal neurons from control and IN-*PS* cDKO mice at a holding potential of − 70 mV in the presence of blockers of AMPA (10 µM NBQX) and NMDA (50 µM APV) receptors. **b** Cumulative inter-event interval (Control: 2033 events in 12 slices, cDKO: 2791 events in 13 slices) and amplitude (Control: 291 events in 12 slices, cDKO: 483 events in 13 slices) histograms of sIPSCs recorded in slices from control and IN-*PS* cDKO mice, showing a reduction in sIPSC frequency but no change in sIPSC amplitude from IN-*PS* cDKO mice. **c** Statistical analysis indicates that IN-*PS* cDKO neurons exhibit significantly reduced sIPSC frequency (Control: 4.39 ± 0.25 Hz, cDKO: 2.86 ± 0.27 Hz; p = 0.0004, unpaired *t*-test) but unchanged amplitude (Control: 63.3 ± 1.9 pA, cDKO: 59.0 ± 2.7 pA; p = 0.22, unpaired *t*-test). **d** Schematic drawing shows mono-synaptic fast GABAergic inhibition of CA1 pyramidal neurons. **e** Input/output relations of evoked GABA_A_R IPSCs in slices from control and IN-*PS* cDKO mice. The evoked IPSCs were elicited by the stimulation electrode placed close to the recording electrode in the CA1 stratum pyramidale and recorded at a holding potential of − 70 mV with Cs^+^-based pipette solution containing high [Cl^−^]. The IPSC amplitude is plotted as a function of stimulation intensity. IN-*PS* cDKO neurons show reduction of evoked IPSC amplitudes (F_1, 22_ = 5.02, p = 0.03, two-way ANOVA). All data represent mean ± SEM (* p < 0.05, *** p < 0.001). The number of neurons/mice used in each experiment is shown in parentheses

**Fig. 2:**
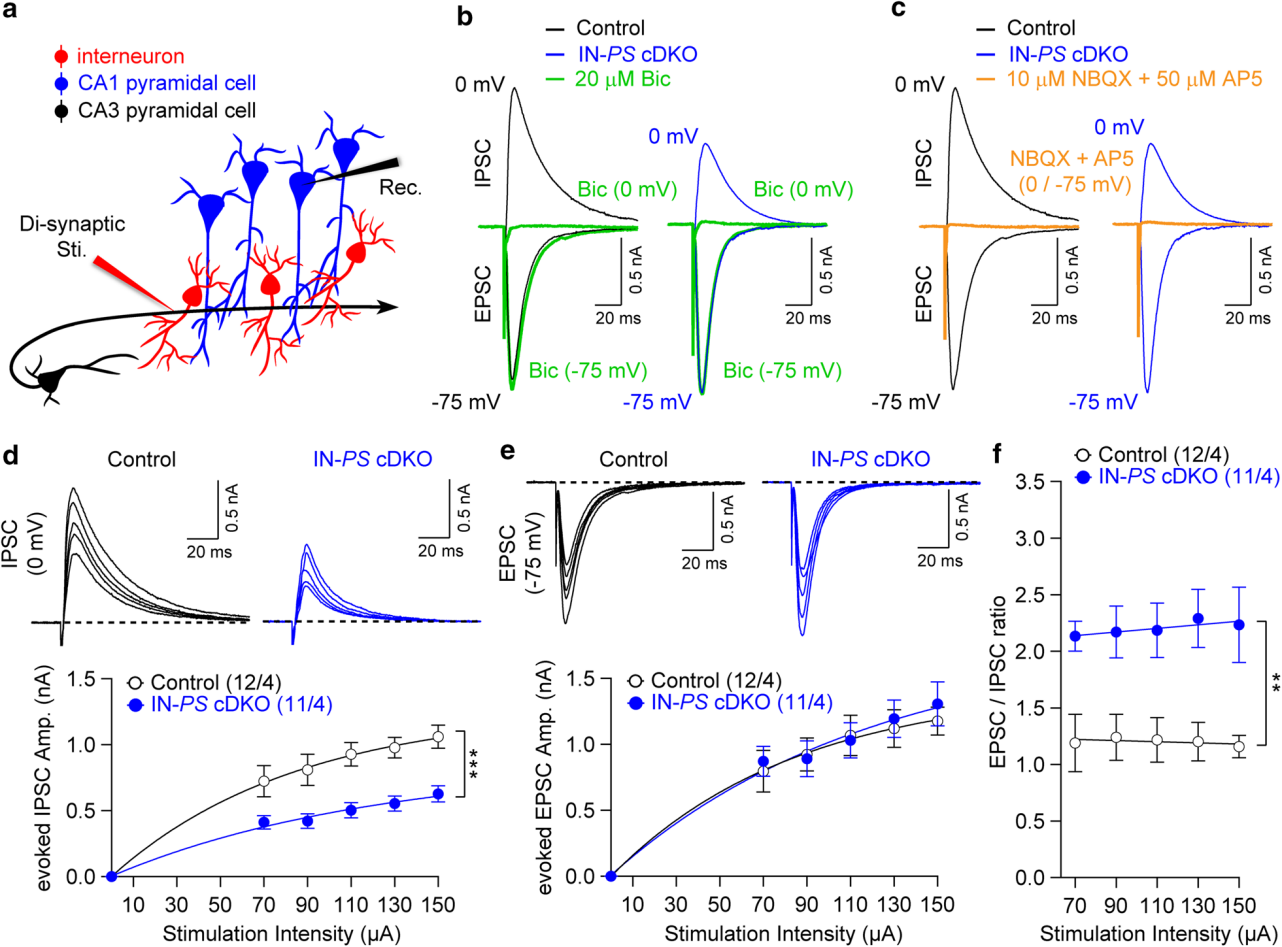
Reduced feed-forward inhibition in hippocampal CA1 neurons of IN-*PS* cDKO mice. **a** Schematic drawing shows the circuitry for bi-phasic synaptic responses in CA1 pyramidal neurons triggered by the SC stimulation. **b** Representative bi-phasic synaptic responses in CA1 pyramidal neurons. Bi-phasic responses were obtained by placing the stimulation electrode away from the recording electrode in the CA1 stratum pyramidale, and IPSCs were recorded at a holding potential of 0 mV and EPSCs were recorded at a holding potential of − 75 mV with low [Cl^−^] Cs^+^-based pipette solution. Synaptic currents recorded at 0 mV were completely blocked by GABA_A_ receptor antagonist bicuculline (20 µM, Bic; green trace at 0 mV). However, the peak amplitude of synaptic currents at − 75 mV was not affected by bicuculline (green trace at − 75 mV). **c** Both IPSC and EPSC (recorded at 0 mV and − 75 mV, respectively) were blocked by NBQX (10 µM) and AP5 (50 µM) (orange traces at 0 mV or − 75 mV). **d** Evoked feed-forward di-synaptic IPSCs in slices from control and IN-*PS* cDKO mice at different stimulation intensities. IN-*PS* cDKO neurons show reduction of evoked IPSC amplitudes (F_1, 21_ = 18.58, p = 0.0003, two-way ANOVA). **e** Normal input/output relations for evoked glutamatergic EPSCs obtained from SC stimulation in slices from control and IN-*PS* cDKO mice (F_1, 21_ = 0.05, p = 0.83, two-way ANOVA). **f** Summary plot showing the averaged EPSC/IPSC amplitude ratios in CA1 pyramidal neurons. IN-*PS* cDKO neurons exhibit significant increases of the EPSC/IPSC ratio compared to controls (F_1, 21_ = 12.93, p = 0.002, two-way ANOVA). All data represent mean ± SEM (** p < 0.01, *** p < 0.001). The number of neurons/mice used in each experiment is shown in parentheses

**Fig. 3:**
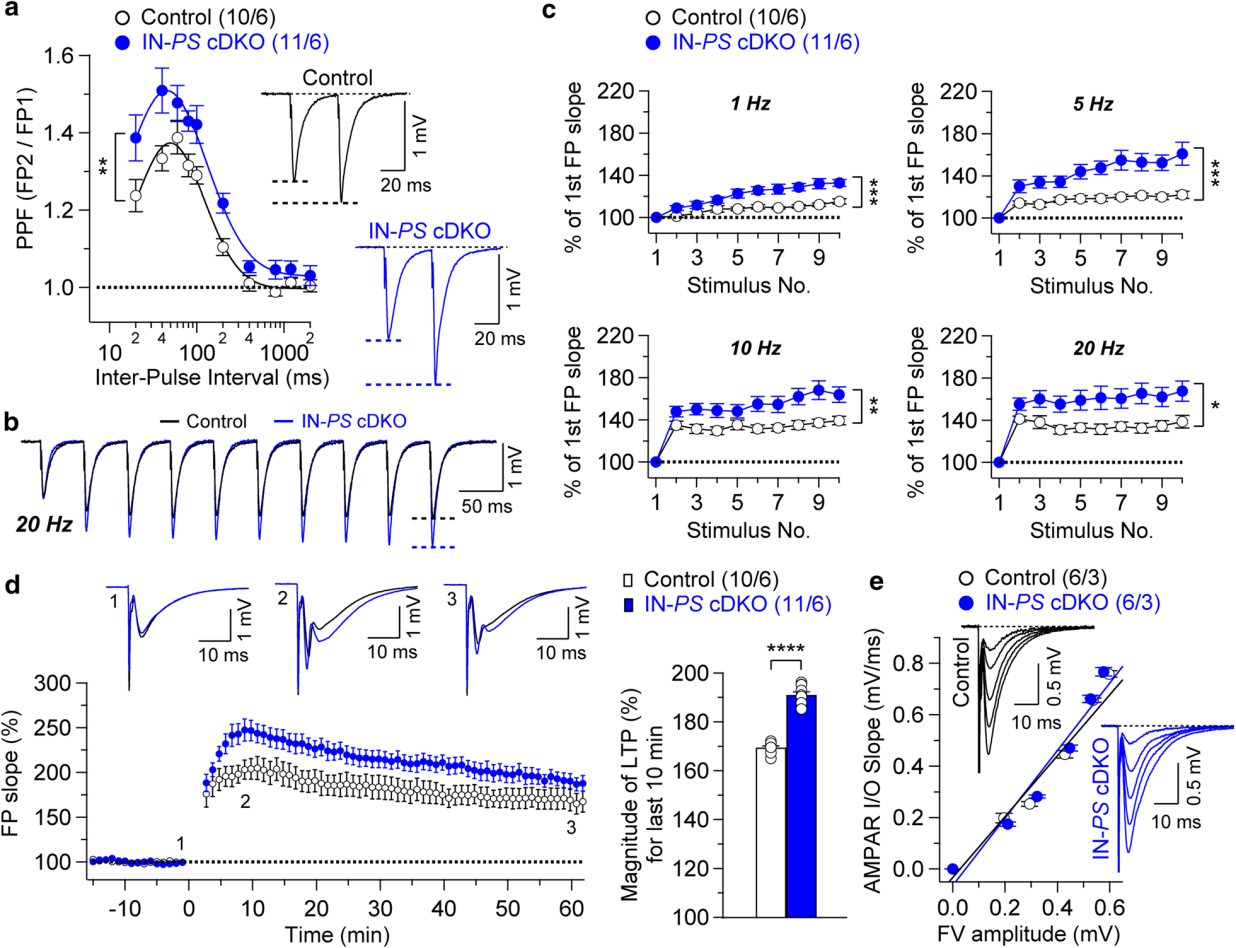
Enhanced synaptic plasticity in the hippocampal Schaffer collateral-CA1 pathway of IN-*PS* cDKO mice. **a** Enhanced PPF in IN-*PS* cDKO mice. Averaged PPF is plotted as a function of the inter-stimulus intervals (20-2000 ms). PPF is higher in IN-*PS* cDKO mice relative to littermate controls (F_1, 19_ = 8.74; p < 0.01, two-way ANOVA). Insets are representative fEPSP traces evoked by two consecutive stimuli with a 40 ms inter-pulse interval. **b** Superimposed fEPSP traces of frequency facilitation elicited by 20 Hz stimulus train show greater enhancement in IN-*PS* cDKO mice relative to controls. **c** Summary graphs show that synaptic facilitation elicited by stimulus trains is enhanced, and that the enhancement is greater in IN-*PS* cDKO mice relative to controls (1 Hz: F_1, 19_ = 21.15, p < 0.001; 5 Hz: F_1, 19_ = 16.35, p < 0.001; 10 Hz: F_1, 19_ = 8.75, p < 0.01; 20 Hz: F_1, 19_ = 7.41, p < 0.05; two-way ANOVA). **d** Enhanced LTP induced by 5 trains of TBS in IN-*PS* cDKO mice. Superimposed traces are averages of four consecutive responses 1 min before (1), 7 min and 60 min after (2, 3) TBS induction. Summary graph shows the magnitude of LTP measured during the last 10 min post-induction (51–60 min) in control and IN-*PS* cDKO hippocampal slices. **e**Normal AMPA receptor-mediated synaptic transmission in IN-*PS* cDKO mice. The lines represent the best linear regression fit. The input/output slopes are similar between control (y = 1.27x, R^2^ = 0.96) and IN-*PS* cDKO (y = 1.32x, R^2^ = 0.94) mice (p = 0.46; linear regression). All data represent mean ± SEM (* p < 0.05, ** p < 0.01, *** p < 0.001, **** p < 0.0001). The number of slices/mice used in each experiment is shown in parentheses

**Fig. 4:**
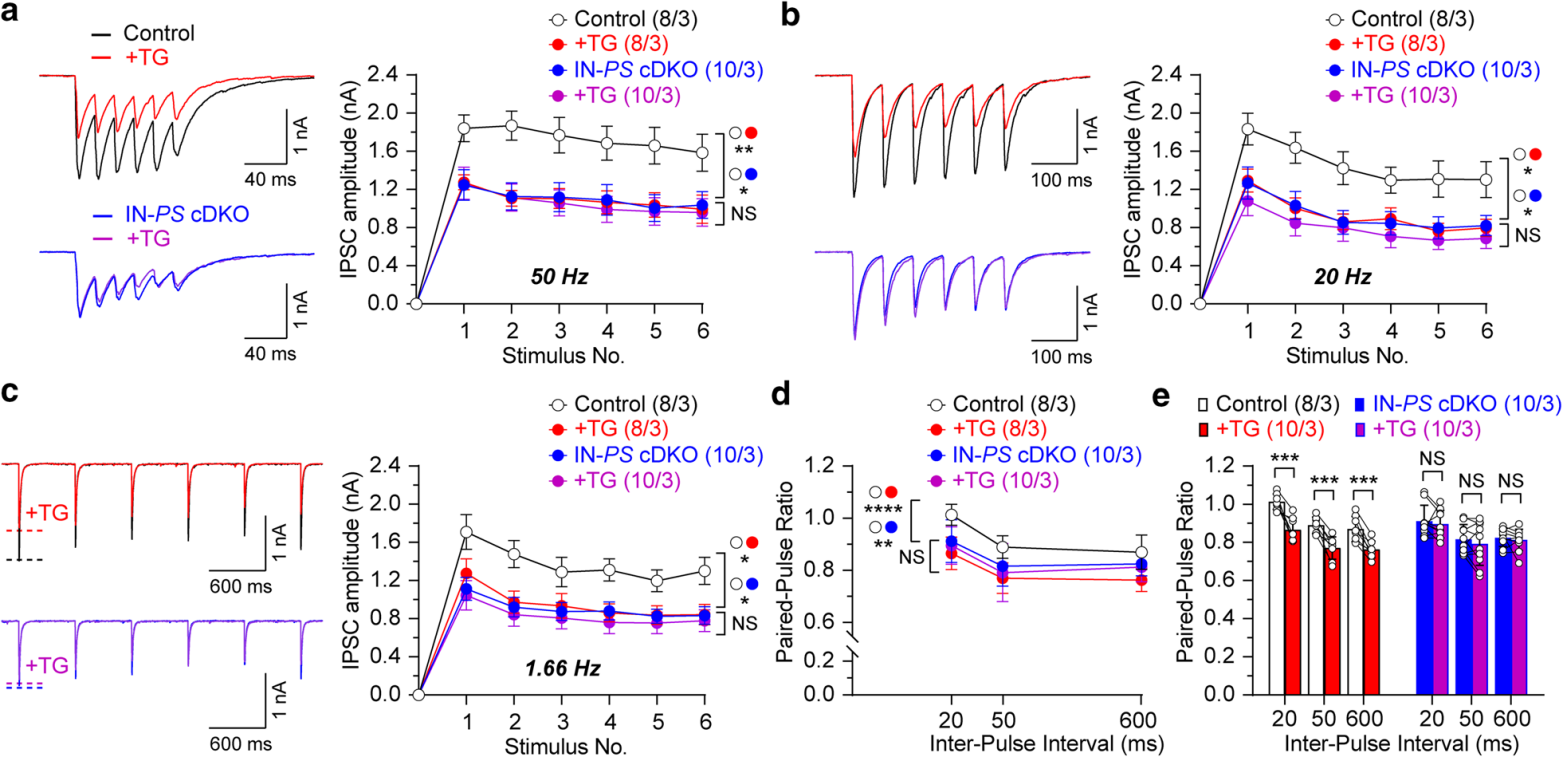
Impairment of ER Ca^2+^ homeostasis in hippocampal CA1 neurons of IN-*PS* cDKO mice. Representative IPSCs evoked by 6 repetitive stimulation pulses at 50 Hz (**a**), 20 Hz (**b**) and 1.66 Hz (**c**) recorded before and after addition of the SERCA inhibitor, TG (2 μM for 30 min). Evoked IPSCs obtained from mono-synaptic stimulation in slices from control and IN-*PS* cDKO mice at 110 µA stimulation intensity. Summary graphs show that GABAergic synaptic responses are decreased in IN-*PS* cDKO neurons relative to controls (50 Hz: p = 0.012; 20 Hz: p = 0.019; 1.66 Hz: p = 0.011; two-way ANOVA). The TG treatment of slices from control mice results in reduced IPSC amplitudes of CA1 neurons (50 Hz: p = 0.007; 20 Hz: p = 0.013; 1.66 Hz: p = 0.032; two-way ANOVA), whereas TG treatment of slices from IN-*PS* cDKO mice fails to reduce the amplitude of IPSCs triggered by stimulus trains. **d** Reduced PPR in IN-*PS* cDKO mice relative to controls (p = 0.004, two-way ANOVA). The TG treatment of slices from control mice results in reduced PPR (p < 0.0001, two-way ANOVA), whereas TG treatment of slices from IN-*PS* cDKO mice fails to reduce the PPR. **e** Summary graphs display individual values of PPR before and after TG application. PPR is significantly decreased after 30 min treatment of TG in control neurons (20 ms: p = 0.0008; 50 ms: p = 0.0006; 600 ms: p = 0.0007; paired *t*-test), whereas the effect of TG treatment on PPR in IN-*PS* cDKO neuron is not statistically significant (20 ms: p = 0.51; 50 ms: p = 0.29; 600 ms: p = 0.52; paired *t*-test). All data represent mean ± SEM (* p < 0.05, ** p < 0.01, *** p < 0.001, **** p < 0.0001; NS: not significant). The number of neurons/mice used in each experiment is shown in parentheses

Statistical analysis was conducted using Student’s *t*-test (Figs. 1c, 3d, 4e, Additional file 1: Fig. 1C) or two-way ANOVA followed up by Bonferroni multiple comparison test (Figs. 1e, 2d–f, 3a, 3c, 4a–d) and linear regression fit (Fig. 3e). All statistical comparisons were performed on data from ≥ 3 biologically independent samples and replicated on different experimental days. Significance is shown as *p < 0.05, **p < 0.01, ***p < 0.001, ****p < 0.0001, or NS (not significant).

## Results

### Decreased GABAergic synaptic responses in IN-*PS* cDKO mice

Although GABAergic interneurons constitute a small percentage of hippocampal neurons (~ 10%), they are fundamentally important for the regulation of hippocampal network’s functions [25, 26]. To investigate the normal physiological role of PS in interneurons and how it modulates excitatory neurotransmission in the hippocampal networks, we recorded GABA_A_ receptor-mediated synaptic responses in CA1 neurons of IN-*PS* cDKO and control mice. First, we found that the frequency of spontaneous inhibitory postsynaptic currents (sIPSCs) is reduced in IN-*PS* cDKO (2.86 ± 0.27 Hz), compared to littermate controls (4.39 ± 0.25 Hz; Fig. 1a–c, p = 0.0004, unpaired *t*-test), whereas the amplitude of sIPSCs is similar between IN-*PS* cDKO (59.0 ± 2.7 pA) and control (63.3 ± 1.9 pA) neurons (Fig. 1a–c; p = 0.22, unpaired *t*-test).

We then directly stimulated interneurons, triggering mono-synaptic fast GABAergic inhibitory synaptic responses in CA1 pyramidal neurons. In these experiments, we recorded evoked IPSCs in CA1 neurons in the presence of blockers of AMPA (10 µM NBQX) and NMDA (50 µM APV) receptors at a holding potential of − 70 mV with high [Cl^−^] intrapipette solution. The stimulation electrode was placed close to the recording electrode in the CA1 stratum pyramidale area, thus allowing direct activation of local circuit interneurons (Fig. 1d). We found that the functional efficacy of inhibition, as assayed with input/output relations for evoked GABA_A_ receptor-mediated IPSCs, is lowered in IN-*PS* cDKO neurons compared to littermate control mice (Fig. 1e; F_1, 22_ = 5.02, p = 0.03, two-way ANOVA). These results indicate that GABA_A_ receptor-mediated inhibition in CA1 area of the hippocampus is impaired in the absence of PS in interneurons.

### Reduced Di-synaptic GABAergic responses in IN-*PS* cDKO mice

Projections from hippocampal CA3 axons form direct excitatory connections on interneurons producing di-synaptic IPSCs in CA1 neurons (Fig. 2a). Thus, stimulation of CA3 axon fibers induces bi-phasic synaptic responses in CA1 pyramidal neurons, comprised of early mono-synaptic EPSCs recorded at a holding potential of − 75 mV and delayed IPSCs recorded at a holding potential of 0 mV when intrapipette solution contains a low concentration of Cl^−^. The GABA_A_ receptor antagonist bicuculline (20 μM) suppressed synaptic currents recorded at 0 mV, whereas peak amplitudes of synaptic currents at − 75 mV were unaffected, indicating the lack of cross-contamination between IPSCs and EPSCs under our recording conditions (Fig. 2b). The joint application of the AMPAR and NMDAR antagonists, (10 μM NBQX and 50 μM AP5, respectively) inhibited both IPSCs and EPSCs (Fig. 2c), providing evidence of the di-synaptic nature of IPSCs recorded in CA1 neurons. Consistent with the di-synaptic nature of recorded IPSCs, we found that their synaptic delays (measured from the onset of stimulation to the onset of the IPSC) are significantly longer compared to the delays induced by mono-synaptic stimulation (see Additional file 1: Fig. 1A–C). The strength of di-synaptic inhibition in CA1 neurons, as assayed with input/output relations for evoked IPSCs obtained from SC stimulation, is markedly reduced in IN-*PS* cDKO mice compared to littermate controls (Fig. 2d; F_1, 21_ = 18.58, p = 0.0003, two-way ANOVA). The input/output relations for evoked EPSCs in the same recordings are not significantly different between control and IN-*PS* cDKO mice (Fig. 2e; F_1, 21_ = 0.05, p = 0.83, two-way ANOVA). These results provide further evidence that GABAergic synaptic responses are impaired in the absence of PS in interneurons. Specifically, activation of interneurons by CA3 axon fibers results in diminished feedforward inhibition of CA1 neurons in IN-*PS* cDKO mice. Thus, PS appears to regulate excitation/inhibition balance (EPSC/IPSC ratio; Fig. 2f; F_1, 21_ = 12.93, p = 0.002, two-way ANOVA) in the SC-CA1 projections, possibly affecting the signal flow in hippocampal microcircuits.

### Enhanced synaptic plasticity in IN-*PS* cDKO mice

We then examined both short-term and long-term synaptic plasticity in IN-*PS* cDKO and littermate control mice to further determine the functional consequences of PS inactivation in interneurons. We evaluated two forms of short-term synaptic plasticity, paired-pulse facilitation (PPF) and frequency facilitation. PPF, induced by two paired stimuli delivered at inter-pulse intervals ranging from 20 to 2000 ms, is significantly higher in IN-*PS* cDKO hippocampal slices relative to controls, suggesting decreased probability of neurotransmitter release at SC-CA1 synapses in the absence of PS in interneurons (Fig. 3a; F_1, 19_ = 8.74, p = 0.008, two-way ANOVA). Consistent with these results, frequency facilitation, induced by short trains of presynaptic stimulation (10 pulses), which were delivered at frequencies ranging from 1 to 20 Hz, is also more prominent in IN-*PS* cDKO mice (Fig. 3b and c; 1 Hz: F_1, 19_ = 21.15, p = 0.0002; 5 Hz: F_1, 19_ = 16.35, p = 0.0008; 10 Hz: F_1, 19_ = 8.75, p = 0.0084; 20 Hz: F_1, 19_ = 7.41, p = 0.014; two-way ANOVA).

We next examined the effects of PS inactivation in inhibitory neurons on LTP at SC-CA1 synapses. LTP was induced by five trains of theta burst stimulation (TBS) and the potentiation was quantified by measuring the changes in the initial slope of the evoked fEPSPs. Notably, we found a greater LTP at SC-CA1 synapses in IN-*PS* cDKO mice. The magnitude of LTP measured during the last 10 min post-induction (51–60 min) after TBS in IN-*PS* cDKO mice is significantly higher (191.1 ± 1.1%) relative to control mice (Fig. 3d; 169.5% ± 0.6%; p < 0.0001, unpaired *t*-test). These results suggest that inactivation of PS may enhance LTP induction via reductions in the function of GABAergic interneurons.

To determine whether the observed changes in synaptic plasticity may be caused by the alterations of excitatory neurotransmission, we examined AMPA receptor-mediated input/output (I/O) curves at the SC-CA1 synapses. We found that AMPA receptor-mediated I/O relationships obtained by plotting the amplitude of fiber volley (FV) versus the field excitatory postsynaptic potentials (fEPSPs) slope in the presence of blockers of NMDA (50 µM APV) and GABA_A_ receptors (10 µM bicuculline) are similar between IN-*PS* cDKO and control mice (Fig. 3e; p = 0.46, linear regression). Thus, normal AMPA receptor-mediated basal synaptic transmission in IN-*PS* cDKO neurons may be maintained due to the normal levels of PS1 expressed in excitatory neurons lacking Cre expression.

Together, these results show synaptic imbalance in IN-*PS* cDKO mice and indicate that PS in inhibitory neurons are required for normal short-term and long-term synaptic plasticity at hippocampal SC-CA1 synapses.

### Impaired ER Ca^2+^ homeostasis in IN-*PS* cDKO mice

To determine whether the reduction of GABAergic synaptic responses triggered by activation of interneurons by glutamatergic SC inputs in IN-*PS* cDKO mice is at least in part due to ER Ca^2+^ dysregulation in interneurons, we examined the magnitude of monosynaptic IPSC changes in the course of repetitive stimulation in the absence or presence of thapsigargin (TG; 2 µM, for 30 min), which is a specific inhibitor of SERCA [27], at CA1 neurons of IN-*PS* cDKO and control mice. We found that the amplitude of IPSCs induced by 6 stimuli applied at frequencies ranging from 1.66 to 50 Hz is decreased during the train in IN-*PS* cDKO mice compared to controls (Fig. 4a–c; 50 Hz: F_1, 16_ = 7.93, p = 0.012; 20 Hz: F_1, 16_ = 6.80, p = 0.019; 1.66 Hz: F_1, 16_ = 8.36, p = 0.011; two-way ANOVA). The application of TG (20 μM) results in significant reductions of IPSC amplitude in control mice (Fig. 4a–c; 50 Hz: F_1, 14_ = 10.07, p = 0.007; 20 Hz: F_1, 14_ = 7.99, p = 0.013; 1.66 Hz: F_1, 14_ = 5.64, p = 0.032; two-way ANOVA), whereas TG treatment of slices from IN-*PS* cDKO mice fails to reduce the amplitude of IPSCs triggered by stimulus trains (Fig. 4a–c; 50 Hz: F_1, 18_ = 0.05, p = 0.83; 20 Hz: F_1, 18_ = 0.60, p = 0.45; 1.66 Hz: F_1, 18_ = 0.24, p = 0.63; two-way ANOVA). These results indicate that the effects of SERCA blockade on inhibitory neurons are occluded in IN-*PS* cDKO mice. Furthermore, IPSC amplitudes in IN-*PS* cDKO neurons are similar to those in control neurons following TG treatment (Fig. 4a–c; 50 Hz: F_1, 16_ = 0.001; p = 0.97, 20 Hz: F_1, 16_ = 0.00003, p = 0.99; 1.66 Hz: F_1, 16_ = 0.099, p = 0.76; two-way ANOVA), suggesting that depletion of ER Ca^2+^ in interneurons has the same effect on GABAergic responses as PS inactivation.

We then evaluated the magnitude of paired-pulse depression of IPSCs at the studied synapses by examining IPSCs induced by paired-pulse stimulation at 20, 50 and 600 ms inter-stimulus intervals. We found that paired-pulse ratio (PPR) is significantly lower in IN-*PS* cDKO mice relative to controls (Fig. 4d; F_1, 16_ = 11.73, p = 0.004, two-way ANOVA). Consistent with the alterations of IPSC amplitudes elicited by repetitive stimulation, PPR is dramatically reduced in control neurons (Fig. 4d, e; F_1, 14_ = 76.73.64, p < 0.0001, two-way ANOVA) but not significantly altered in IN-*PS* cDKO neurons following TG treatment (Fig. 4d; F_1, 18_ = 0.40, p = 0.54, two-way ANOVA). These results show that inactivation of PS mimics the effects of blockade of SERCA, suggesting that disrupted ER Ca^2+^ homeostasis in inhibitory interneurons of IN-*PS* cDKO mice may contribute to the observed impairments in sort-term synaptic plasticity.

## Discussion

In the current study, we investigate the role of PS in interneurons at the local circuit of the hippocampal SC-CA1 pathway using IN-*PS* cDKO mice, in which PS is selectively inactivated by Cre recombinase expressed under control of the endogenous *GAD2* promoter [24]. We chose to analyze IN-*PS* cDKO mice at the age of 2 months before they start to exhibit reduced body weight and enhanced mortality [24]. Our whole-cell and field recordings showed that PS inactivation results in impaired GABAergic inhibition and hyperactivity of CA1 neurons at the SC synapse of IN-*PS* cDKO mice. We previously reported that PS plays an essential role in the regulation of short-term plasticity, long-term potentiation and neurotransmitter release in the hippocampal SC and mossy fiber (MF) pathways of excitatory neuron-specific *PS* cDKO (EX-*PS* cDKO) mice [2, 4, 6, 8, 9]. Thus, it would be of great interest to perform similar electrophysiological analysis to determine the consequences of selective PS inactivation in interneurons on inhibitory and excitatory synaptic responses in the hippocampal local network.

To determine the normal physiological role of PS in interneurons and how selective PS inactivation in inhibitory neurons influences the excitatory neurotransmission in the hippocampal network, we examined GABA_A_ receptor-mediated synaptic responses in CA1 neurons. We found that the frequency of sIPSCs is markedly reduced in IN-*PS* cDKO mice compared to littermate controls, whereas the amplitude of sIPSCs is similar between IN-*PS* cDKO and control neurons (Fig. 1a–c). These results indicate that inactivation of PS in interneurons causes impaired presynaptic GABAergic signaling.

To examine further GABAergic neurotransmission in IN-*PS* cDKO mice, we directly stimulated interneurons, thus triggering mono- or di-synaptic GABAergic inhibitory responses. We found significant decreases of functional efficacy of inhibition at the level of input/output relations for evoked GABA_A_ receptor-mediated IPSCs in CA1 neurons of IN-*PS* cDKO mice (Fig. 1e and 2d), whereas input/output relations for evoked EPSCs in the same recordings are similar between the genotypic groups (Fig. 2e). These results imply that GABA_A_ receptor-mediated inhibition in hippocampal area CA1 is reduced in the absence of PS in interneurons. Accordingly, IN-*PS* cDKO mice exhibit upward-shifted EPSC/IPSC ratio (Fig. 2f), suggesting that PS inactivation in the interneuron may cause an abnormal hyperactive network activity. Moreover, both short- and long-term synaptic plasticity (including PPF, frequency facilitation and LTP) at the SC-CA1 synapse are enhanced in IN-*PS* cDKO mice. Consistent with these findings, previous studies showed that TBS-induced LTP at SC-CA1 synapses is enhanced upon GABAergic inhibition at local circuits [28, 29]. Thus, the disruption of the balance between synaptic excitation and inhibition may result in impaired synaptic function and deficits in learning and memory.

We previously reported that ryanodine receptor (RyR)-mediated Ca^2+^ release from the ER is impaired in EX-*PS* cDKO mice, and depletion of ER Ca^2+^ release mimics and occludes the Ca^2+^ defects and dysfunction of presynaptic short-term plasticity observed in EX-*PS* cDKO mice [6, 8]. In the present study, we found similarly that depletion of ER Ca^2+^ by thapsigargin results in reduction of IPSC amplitude in control neurons, and that thapsigargin treatment does not cause further reductions of the IPSC amplitude in IN-*PS* cDKO neurons (Fig. 4). Thus, these findings indicate that inactivation of PS in interneurons also mimics and occludes the effects of blockade of SERCA, suggesting that disrupted ER Ca^2+^ homeostasis in interneurons may contribute to the observed synaptic dysfunction, and that PS regulates intracellular Ca^2+^ homeostasis in both excitatory and inhibitory neurons.

In hippocampal area CA1, there are several types of GABAergic interneurons based on their expression of parvalbumin (PV), somatostatin (SST), or cholecystokinin, and their axonal arborization density and long‐range projections [30]. There are at least 12 distinct interneuron subtypes that innervate mainly or exclusively onto the dendrites of CA1 pyramidal neurons [31], though the specific function of these dendrite-innervating interneurons in the hippocampal network is not fully understood. Interestingly, loss of PV- and SST-expressing interneurons has been reported in the hippocampus and entorhinal cortex in AD patients [32–36]. Therefore, it would be interesting to perform further studies to determine the role of PS in specific interneuronal subtypes and investigate the molecular mechanism by which PS regulates fast GABAergic neurotransmission in the hippocampus.

## Supplementary Information

**Additional file 1: Fig. S1.** The synaptic latency of the IPSC triggered by the SC stimulation is significantly longer 6 compared to the latency of the IPSC induced by direct stimulation resulting in mono-synaptic responses. **A**) *Left*: Superimposed examples of mono-synaptic IPSCs in control and IN-*PS* cDKO neurons. Scale bar: 20 ms, 1 nA. *Right*: IPSCs are shown on an expanded time scale. The arrowhead indicates delays from the onset of stimulation to the onset of the IPSC. Scale bar: 1 ms, 1 nA. **B**) *Left*: Superimposed examples of di-synaptic IPSCs in control and IN-*PS* cDKO neurons. Scale bar: 20 ms, 1 nA. *Right*: IPSCs are shown on an expanded time scale. The arrowhead indicates delays from the onset of stimulation to the onset of the IPSC. Scale bar: 1 ms, 1 nA. **C**) Bar graphs showing the mean values of delays of mono- and di-synaptic IPSCs. There is no significant difference between control and IN-*PS* cDKO neurons in delays of mono-synaptic (p = 0.96) and di-synaptic IPSC responses (p = 0.81, unpaired *t*-test). However, the delays of di-synaptic IPSC responses are markedly longer than the delays of mono-synaptic IPSC responses (Control: p < 0.0001; IN-*PS* cDKO: p < 0.0001; unpaired *t*-test). All data represent mean ± SEM (**** p < 0.0001; NS: not significant). The number of neurons/mice in each experimental group is shown in parentheses.

## Data Availability

The datasets generated and/or analyzed during the current study are available from the corresponding author on reasonable request.

## Abbreviations

ACSF: Artificial cerebrospinal fluid
AD: Alzheimer’s disease
AMPA: α-Amino-3-hydroxy-5-methyl-4-isoxazolepropionic acid
AP: Action potential
CA1: Cornu Ammonis 1 area of hippocampus
CA3: Cornu Ammonis 3 area of hippocampus
Cl^−^: Chloride ion
EPSC: Excitatory postsynaptic current
ER: Endoplasmic reticuli
EX-*PS* cDKO: Excitatory neuron-specific *Presenilin* conditional double knockout
fEPSP: Field excitatory postsynaptic potential
GABA: Gamma-aminobutyric acid
GABA_A_ receptor: Gamma-aminobutyric acid receptor subtype A
GAD2: Glutamate decarboxylase2
IN-*PS* cDKO: Inhibitory neuron-specific *Presenilin* conditional double knockout
I/O: Input/output
IPSC: Inhibitory postsynaptic current
LTP: Long-term potentiation
MF: Mossy fiber
NMDA: N-methyl-D-aspartate
PPF: Paired-pulse facilitation
PPR: Paired-pulse ratio
PS: Presenilins
PV: Parvalbumin
RyR: Ryanodine receptor
SC: Schaffer collateral
SERCA: Sarcoendoplasmic reticulum Ca^2^^+^ ATPase
sIPSCs: Spontaneous inhibitory postsynaptic currents
SST: Somatostatin
TBS: Theta burst stimulation
TG: Thapsigargin
