# Supplementary material for: Inactivation of Presenilin in inhibitory neurons results in decreased GABAergic responses and enhanced synaptic plasticity
Source: Mol Brain. 2021 May 25;14:85. doi: 10.1186/s13041-021-00796-5 (PMC8152317; doi:10.1186/s13041-021-00796-5)
Supplement: Supplementary file 1 — Additional file 1: Fig. S1. The synaptic latency of the IPSC triggered by the SC stimulation is significantly longer 6 compared to the latency of the IPSC induced by direct stimulation resulting in mono-synaptic responses. A) Left: Superimposed examples of mono-synaptic IPSCs in control and IN-PS cDKO neurons. Scale bar: 20 ms, 1 nA. Right: IPSCs are shown on an expanded time scale. The arrowhead indicates delays from the onset of stimulation to the onset of the IPSC. Scale bar: 1 ms, 1 nA. B) Left: Superimposed examples of di-synaptic IPSCs in control and IN-PS cDKO neurons. Scale bar: 20 ms, 1 nA. Right: IPSCs are shown on an expanded time scale. The arrowhead indicates delays from the onset of stimulation to the onset of the IPSC. Scale bar: 1 ms, 1 nA. C) Bar graphs showing the mean values of delays of mono- and di-synaptic IPSCs. There is no significant difference between control and IN-PS cDKO neurons in delays of mono-synaptic (p = 0.96) and di-synaptic IPSC responses (p = 0.81, unpaired t-test). However, the delays of di-synaptic IPSC responses are markedly longer than the delays of mono-synaptic IPSC responses (Control: p < 0.0001; IN-PS cDKO: p < 0.0001; unpaired t-test). All data represent mean ± SEM (**** p < 0.0001; NS: not significant). The number of neurons/mice in each experimental group is shown in parentheses. [file 13041_2021_796_MOESM1_ESM.pdf]

Additional file 1

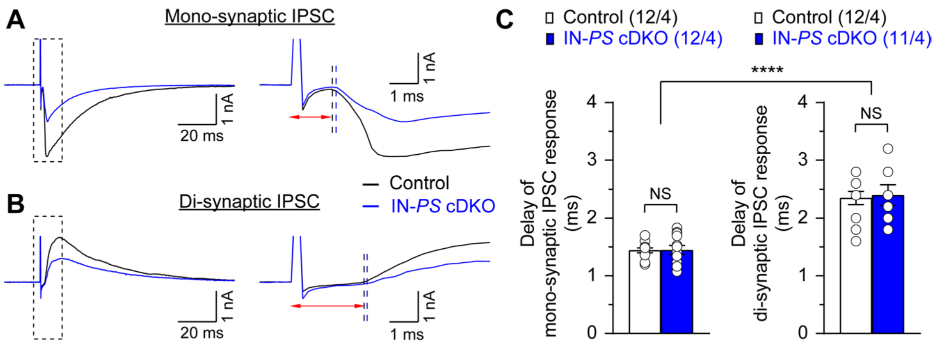

Additional Fig. 1 The synaptic latency of the IPSC triggered by the SC stimulation is significantly longer compared to the latency of the IPSC induced by direct stimulation resulting in mono-synaptic responses. **A**) *Left*: Superimposed examples of mono-synaptic IPSCs in control and IN-*PS* cDKO neurons. Scale bar: 20 msec, 1 nA. *Right*: IPSCs are shown on an expanded time scale. The arrowhead indicates delays from the onset of stimulation to the onset of the IPSC. Scale bar: 1 msec, 1 nA. **B**) *Left*: Superimposed examples of di-synaptic IPSCs in control and IN-*PS* cDKO neurons. Scale bar: 20 msec, 1 nA. *Right*: IPSCs are shown on an expanded time scale. The arrowhead indicates delays from the onset of stimulation to the onset of the IPSC. Scale bar: 1 msec, 1 nA. **C**) Bar graphs showing the mean values of delays of mono- and di-synaptic IPSCs. There is no significant difference between control and IN-*PS* cDKO neurons in delays of mono-synaptic ($p = 0.96$) and di-synaptic IPSC responses ($p = 0.81$, unpaired *t*-test). However, the delays of di-synaptic IPSC responses are markedly longer than the delays of mono-synaptic IPSC responses (Control: $p < 0.0001$; IN-*PS* cDKO: $p < 0.0001$; unpaired *t*-test). All data represent mean ± SEM (**** $p < 0.0001$; NS: not significant). The number of neurons/mice in each experimental group is shown in parentheses.
